# Visual Analytics: A Method to Explore Natural Histories of Oral Epithelial Dysplasia

**DOI:** 10.3389/froh.2021.703874

**Published:** 2021-08-05

**Authors:** Stan Nowak, Miriam Rosin, Wolfgang Stuerzlinger, Lyn Bartram

**Affiliations:** ^1^School of Interactive Arts and Technology, Simon Fraser University, Burnaby, BC, Canada; ^2^BC Oral Cancer Prevention Program, Cancer Control Research, BC Cancer, Vancouver, BC, Canada; ^3^Department of Biomedical Physiology and Kinesiology, Simon Fraser University, Burnaby, BC, Canada

**Keywords:** oral cancer, visual analytics, artificial intelligence, low-grade oral dysplasia, prevention

## Abstract

Risk assessment and follow-up of oral potentially malignant disorders in patients with mild or moderate oral epithelial dysplasia is an ongoing challenge for improved oral cancer prevention. Part of the challenge is a lack of understanding of how observable features of such dysplasia, gathered as data by clinicians during follow-up, relate to underlying biological processes driving progression. Current research is at an exploratory phase where the precise questions to ask are not known. While traditional statistical and the newer machine learning and artificial intelligence methods are effective in well-defined problem spaces with large datasets, these are not the circumstances we face currently. We argue that the field is in need of exploratory methods that can better integrate clinical and scientific knowledge into analysis to iteratively generate viable hypotheses. In this perspective, we propose that visual analytics presents a set of methods well-suited to these needs. We illustrate how visual analytics excels at generating viable research hypotheses by describing our experiences using visual analytics to explore temporal shifts in the clinical presentation of epithelial dysplasia. Visual analytics complements existing methods and fulfills a critical and at-present neglected need in the formative stages of inquiry we are facing.

## 1. Introduction

The lack of understanding of the natural history of oral cancer is a major barrier to our ability to impactfully intervene early in the disease. As a collective group, clinicians and scientists have followed patients with clinical lesions and dysplastic disease for decades. There are unused files full of text, pictures, and annotations on these patients. In addition, as our capacity to examine biological change underlying time-varying shifts in lesions has accelerated, there is simultaneously additional, increasingly diverse information from scientists coming in. A key missing component in this effort is methods that allow us to frame and utilize such complex and heterogeneous data. They are highly multi-faceted and demand the integration of diverse clinical and scientific knowledge to generate testable hypotheses informed by the most comprehensive understanding of why lesions shift over time and when such changes may be clinically important.

Traditional statistics or the newer machine learning and artificial intelligence (henceforth ML/AI) methods are ill-suited to address many of the immediate challenges faced. The small sample sizes and complexity of clinical datasets limit the types of questions that can be answered. Additionally, these methods generally rely on well-defined and narrow questions. This is appropriate for summative analyses that aim to evaluate specific hypotheses and expectations. Current research, however, is at an exploratory stage. Instead, formative approaches that aim to understand how clinical data might be interrogated, and that support the scientific inductive process of developing, testing, and iterating over a theory are better suited. This requires the integration of expert knowledge into analysis. Data on their own do not offer explanations of why certain patterns or relationships within them exist. From the understanding of procedures involved in data gathering to theories of how observed data relate to underlying biological mechanisms driving dysplastic disease, clinical, and scientific knowledge is key. Unfortunately, statistical and ML/AI methods often require significant training for interpretation and even with sufficient training often remain as difficult-to-understand “black boxes.”

We have faced this problem in British Columbia for some time. The Oral Cancer Prediction Longitudinal (OCPL) study was established over 20 years ago, to follow patients with biopsy-confirmed primary mild and moderate epithelial dysplasia (henceforth low-grade dysplasia, LGD). The presence of epithelial dysplasia is one of the strongest predictors of transformation of LGD to oral cancer; yet there are many unresolved issues around such lesions. The long-term goal of the OCPL study is to use this cohort, with its diverse data on clinical, histologic, and molecular change, and its samples, to help us answer some of the key management questions for these patients: Which of these dysplastic lesions is at risk for progression? Which do we treat, and if we treat, when and how do we do it? There are close to 600 cases in the OCPL study, many with between 10 and 20 years of follow-up, with over 7,000 visits for these patients—a rich resource to identify and study the diverse patterns of temporal change as they occur and look for associations with transformation risk.

The question addressed in this paper is faced by all of us working in this area. How do we deal with this complex and increasingly multi-faceted data pool, especially when dealing with temporal shifts in patient data? How do we use such information to drive meaningful change—to link patterns across data sources and to generate new testable ideas? Where do we begin?

We argue for methods that support the iterative scientific process needed to integrate clinical and mechanistic knowledge. We propose that *visual analytics* (VA) is well-suited to such a niche, providing an approach that can be used to integrate “data-driven” and “knowledge-driven” processes into an iterative analysis that can improve our understanding of the natural history of oral cancer development. In this paper, we describe the challenges of heavily “data-driven” methods and why VA is well suited to complement such methods. We illustrate the value of VA by discussing a simple exploratory visual analysis of lesion shifts in oral dysplasia we conducted using data from the OCPL study.

## 2. Challenges in Heavily Data-Driven Methods

**Figure 1:**
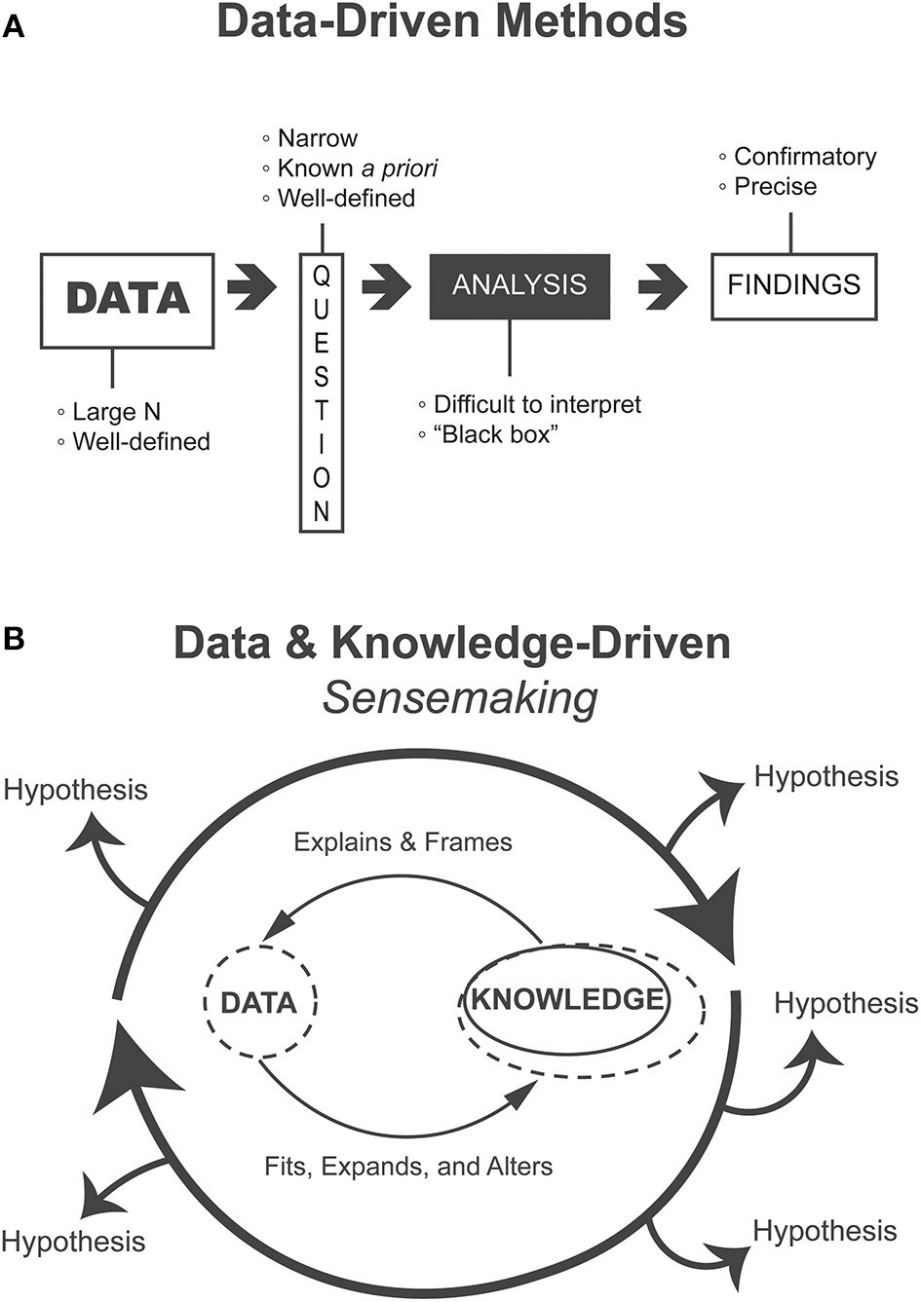
**(A)** Heavily data-driven methods follow a linear flow from data to findings, require voluminous data to address narrow questions that are known ahead of the analysis, and produce confirmatory and precise findings but where analyses may be difficult to interpret “black boxes.” **(B)** Methods that support the data and knowledge-driven process of sensemaking iteratively generate, evaluate, and refine alternative hypotheses. Such methods are appropriate for exploratory and formative analyses.

The rapid change in computational capacity has allowed researchers to increase the volume of data analyzed and to employ sophisticated ML/AI to increasingly complex datasets, which have been inaccessible in the past. Computer vision algorithms can identify cancerous nodules from medical imaging with accuracy sometimes exceeding human experts [1]. Recent preliminary research has also made headway in making these algorithms more interpretable for clinicians [2]. However, state-of-the-art algorithms such as these are applied to *narrow* and *highly specific* tasks and require *large* volumes of highly constrained, well-defined data while relying on a number of assumptions about the statistical properties of these data [3] (Figure 1A).

In contrast, clinical datasets are often complex, heterogeneous, and composed of comparatively much lower volumes of patient data. In addition, patients are diverse and biological processes are ill-understood, and the understanding of how data are gathered is primarily held by clinicians. This creates an ill-defined problem space where the precise questions to ask are not yet known and thus we cannot expect a linear process of well-defined inquiry. Even if there are some well-defined questions, they have not been addressed using existing approaches and progress has been slow. This problem requires iterative and flexible generation and evaluation of practically relevant and knowledge-informed hypotheses (Figure 1B). Presently, natural intelligence is comparatively better than artificial intelligence at dealing with such challenges.

ML/AI algorithms struggle with generalization that goes beyond very constrained problem spaces; they cannot generate causal models of mechanisms underlying the data and translate them to other domains [3]. Generalization involves going beyond what is explicit in data and imagining alternative potential mechanisms of explanation. Counterfactual reasoning, the imagining of alternative events and outcomes, has been the foundation of theories explaining causality [4]. These theories have been integrated into methods used to analyze observational data in epidemiology in the Bradford-Hill criteria [5]. While there is an effort underway to reconcile ML/AI approaches with contemporary causal inference to enable automated discovery of causal structure from data [3], such problems are still largely a human reasoning activity.

## 3. Sensemaking

Sensemaking is a “natural kind of human activity in which large amounts of information about a situation or topic are collected and deliberated upon to form an understanding that becomes the basis of problem-solving and actions” [6]. This activity is often described through the data/frame theory of sensemaking which posits that humans organize knowledge and account for new information using explanatory structures called “frames” [7]. As humans encounter new information through their environment, or in this case visualization systems, the information is matched and fitted to these frames. These frames are then elaborated upon, questioned, rejected, or otherwise manipulated, in our case through the interactive visualization system, in light of any new information. The scientific process of developing, testing, and iterating over theory closely mirrors sensemaking. This flexible way of thinking is what allows humans to meaningfully understand and act in a variety of natural settings such as the exploratory scientific inquiry of data.

An essential component of sensemaking is the generation of alternative hypotheses or interpretations that are flexibly fitted to and altered by data [8]. This process can generate new frames of understanding based on data (data-driven), as well as iterate over existing ones (knowledge-driven) [7]. This iterative fitting and manipulation of data and theory (Figure 1B) integrates human knowledge into analysis without being hampered by the limits of what is explicitly contained in the data. The relatively new field of VA specifically supports such human sensemaking activities.

## 4. Visual Analytics

In scientific domains, visualization is commonly thought of as serving a purely communicative role, primarily supplementing text to emphasize a point. Yet, visualizations, especially interactive ones, can also be used to support a method of analysis. Visual Analytics, the “science of analytical reasoning facilitated by interactive visual interfaces” [9], leverages the strengths of computers to improve human analysis. The aim is to make complex computational processes transparent and empower humans to conduct analysis in an interpretable and accessible way. Rather than replacing ML/AI methods, VA complements these approaches and often integrates them in analysis. Addressing the challenges of interpretability and opening the “black box” of ML/AI algorithms has become a burgeoning area of research in VA [10].

Visualization capitalizes on the innate intelligence of the human visual system. Using external representations as an aid is called “visual thinking” [11]. The human visual system can extract complex statistical patterns from scenes while at the same time linking visual information to high-level cognitive processes. The human visual system is not one passive system, but a number of active systems that can both direct attention to important aspects of data in a bottom-up fashion as well as be directed to search for patterns in a top-down fashion [12]. This interplay between bottom-up (data-driven) and top-down (knowledge-driven) processes in the visual system creates a dynamic interface between humans and data enabling iterative sensemaking processes. This interaction between prior knowledge and perception enables humans to “complete patterns” and derive meaning based on incomplete or uncertain information. The “Gestalt” school of psychology and the concomitant visual Gestalt laws describe these processes [11].

Just as sensemaking in open-ended problem spaces requires the generation and management of alternative hypotheses, VA systems are designed to support alternative visual representations of data to address these hypotheses and help steer the analysis. Some VA systems also incorporate explicit support for managing alternatives [13, 14]. Others have proposed “mixed-initiative” systems that utilize machine learning and data-mining systems that integrate alternative “threads” of analysis as a central system component [15, 16].

VA may seem relatively new, but this approach has already been incorporated in a broad range of domains associated with healthcare and scientific areas. For example, VA has impacted the tracking of disease progression in electronic health records [17], clinical support for blood transfusions [18], decision making in public health [19], genomics [20], chemistry [21], and oncology [22, 23].

## 5. Our Collaborative Project

In this section, we illustrate how VA supports the process of generating, testing, and iterating over alternative hypotheses, using our experiences analyzing a clinical dataset of patients with LGD. We began our analysis around data collected during the examination of clinical lesions. Such assessment is a key initial point in the engagement of a clinician with the patient. It is part of the ascertainment of whether the lesion falls within the “normal” boundaries of change in a tissue, and can thus be triaged back to the community, or instead, requires further follow-up.

Lesions change over time—disappearing, re-appearing, growing in size, altering shape, and changing in texture and appearance. As such, clinical change reflects, in part, alterations occurring at the molecular, cellular, and tissue level. Increasingly there are new developments in clinical approaches and tools used in decision making around lesions. A missing component is our capacity to track changes over time and understand what observable baseline changes, in the absence of intervention, are associated with alterations in progression risk.

Time-based analyses may consider a variety of perspectives or properties of data (e.g., curve fitting, regression, or signal decomposition). When we began these studies, we had no basis to choose any particular type of analysis, and rather than over-constrain the problem-space, we chose to look at sequences which we felt could reveal a variety of patterns in the time-varying data.

Sequences are notoriously challenging for both humans and algorithms to work with [24, 25]. As a preliminary step, we consulted ML experts on an appropriate approach. We employed hidden Markov models (HMMs), a set of algorithms commonly used for mining sequence patterns of biological data [26]. However, the areas where such models have been particularly effective are where the volume of data is quite high, the variety of patterns is relatively low, and the problem space is also relatively constrained. Examples include sequence mining in genetics [27] or protein structure prediction [28]. We discovered early on that we do not have nearly enough data for HMM. Another issue was that our clinical data are relatively complex, reflecting a variety of data-generating processes. The algorithmic output was not strong and we could not find any explanations that could account for the patterns and match existing biological understanding.

We then explored the use of interactive visualizations to analyze these sequences. While algorithmic approaches are often incorporated in VA systems to make sequences and other patterns more tractable [24], for the illustrative purposes of this paper, we will focus on a purely visual approach to highlight how visualizations enable sensemaking and hypothesis generation.

### 5.1. Investigating Shifts in Lesions

**Figure 2:**
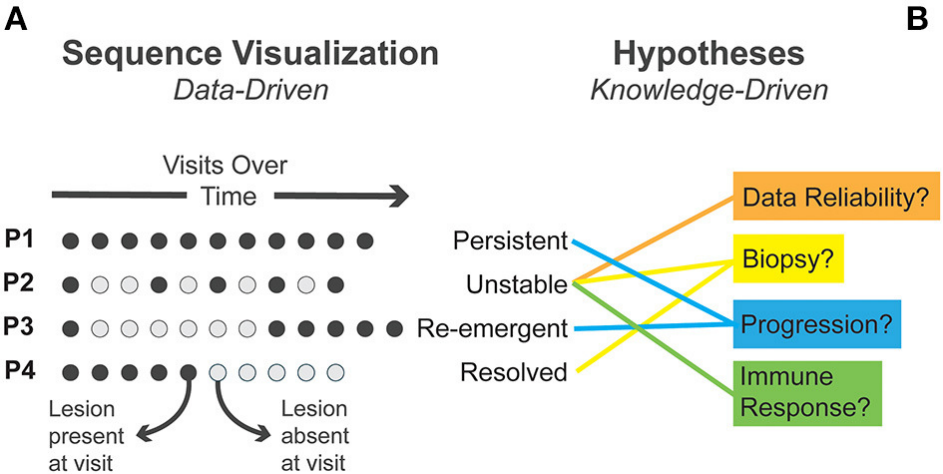
**(A)** Four exemplary sequence patterns in patient visits identified through visual analysis are presented. Circles represent individual visits with time moving left to right. **(B)** Several alternative explanatory mechanisms generated during visual analysis are matched to observed patterns.

We conducted our analysis using simple dot plot visualizations. In Figure 2A, we provide a simplified diagrammatic version of the interactive visualizations we used in analysis to illustrate our process. We identified patterns in the data which indicated potential explanatory mechanisms (Figure 2B). This is an example of how patterns in data (data-driven) can elicit relevant knowledge and thus also influence how important patterns are perceived (knowledge-driven). Drawing on prior domain knowledge, clinical researchers on our team recognized several sequence patterns and iteratively generated alternative hypotheses that could account for such patterns.

We first identified instances where clinical lesions disappeared completely—establishing when lesions were present or absent for each patient (Figure 2). In some patients, the lesion persisted at all time points (termed “persistent lesions”). In others, the lesion disappeared and did not recur during follow-up (termed “resolved”). In some cases, the lesion disappeared early in follow-up and then “re-emerged.” A fourth pattern showed lesions disappearing and reappearing, often multiple times, in an “unstable” fashion.

This process triggered some speculative questions around what could explain these perceived patterns. As a preliminary inquiry, we questioned the reliability of these data as they had not been used in this way before. Clinicians associated with the OCPL study went back to the data to confirm these patterns, using clinical charts, pictures, and the database. As a result of this process and dialogue, several errors in the data were identified and corrected, illustrating the value of visualization at such formative stages.

We also questioned whether shifts in “resolving” and “unstable” lesions associated with small lesion size and excision during biopsy could be confounding the lesion's natural history. We checked. There was no apparent, consistent association with such descriptors. We explored the relationship between these patterns and patient outcomes. Virtually all of the mild or moderate lesions that progressed to severe dysplasia or cancer were persistent lesions. But what intrigued us was the observation that non-progressing lesions fell into two groups: stable, persisting lesions, and unstable lesions, with lesions appearing and disappearing multiple times during follow-up. This generated a series of questions: What was causing the “unstable” phenomena, i.e., what is the underlying biology associated with such change? And did it mean anything for risk or future trajectory of patients? Does it have clinical ramifications/value?

One potential hypothesis is that “unstable” non-progressing lesions could represent those in which protective mechanisms are actively engaged in identifying and removing damaged and genetically altered cells, those with altered signaling pathways, and dysregulated proliferation/differentiation controls. This could involve damage recognition and repair genes, for example, p53-controlled processes, that would trigger events such as senescence or apoptosis. Such changes could also involve cell-cell interactions in the tissue, the local microenvironment, and/or activity of the immune system. These protective systems could switch on and off, as abnormal clones developed and evolved in a lesion. A dysregulation of such systems would result in progression with persistence of the lesions.

The link to the immune system, is particularly attractive, given the rapid evolution of both technology in this area, especially associated with tissue change and risk prediction for cancer development. Recent findings in the esophagus, lung, and oral cavity support the possibility that the immune system is capable of recognizing premalignant lesions and intercepting their progression to cancer [29–32]. Premalignant-specific putative neoantigens have been identified in some such lesions and coupled to tissue infiltration of specific T effector and cytotoxic cells, for example, CD4, CD8, PD-1, and PD-L1 [31]. Finally, early data support the association of alterations to antigen processing and presentation pathways and depletion of innate and adaptive immune cells with premalignant lesions that are more likely to progress. The question is, can we now use this knowledge and our current analysis systems to follow the immune system over time, and look for parallel, concordant alterations in unstable lesions that would support their involvement in temporal shifts?

## 6. Discussion

We have only touched on a small portion of the potential analyses in the research area we have outlined. Even so, our experiences demonstrate the potential for visual analytics to generate and explore new research questions. Conventional methods used in oral oncology research have left many resources, such as complex clinical datasets or the expert knowledge of clinicians, underutilized, and many related questions unasked. It doesn't need to be this way. Using VA allows us to cast a wider net and catch research trajectories that might otherwise remain unexplored. In the context of early detection and prevention of malignant dysplasia, leveraging the data that are already available through clinics has the potential to transform the standard of care.

## Data Availability Statement

The original contributions presented in the study are included in the article/supplementary material, further inquiries can be directed to the corresponding author/s.

## Author Contributions

SN, MR, WS, and LB were all involved in setting the conceptual direction of this work. SN and MR wrote the first draft of this article. WS provided critical feedback and insights and edited the manuscript. All authors contributed to the article and approved the submitted version.

## Conflict of Interest

The authors declare that the research was conducted in the absence of any commercial or financial relationships that could be construed as a potential conflict of interest.

## Publisher's Note

All claims expressed in this article are solely those of the authors and do not necessarily represent those of their affiliated organizations, or those of the publisher, the editors and the reviewers. Any product that may be evaluated in this article, or claim that may be made by its manufacturer, is not guaranteed or endorsed by the publisher.
